# ChiloKey, an interactive identification tool for the geophilomorph centipedes of Europe (Chilopoda, Geophilomorpha)

**DOI:** 10.3897/zookeys.443.7530

**Published:** 2014-09-29

**Authors:** Lucio Bonato, Alessandro Minelli, Massimo Lopresti, Pierfilippo Cerretti

**Affiliations:** 1 Department of Biology, University of Padova, via Ugo Bassi 58B, 35131, Padova, Italy; 2 Centro Nazionale Biodiversità Forestale – Corpo Forestale dello Stato, via Carlo Ederle 16/A, 37128 Verona, Italy; 3 DAFNAE-Entomology, University of Padova, Viale dell’Università 16, 35020, Legnaro, Italy

**Keywords:** Interactive key, identification, morphology, Chilopoda, Geophilomorpha, Europe

## Abstract

ChiloKey is a matrix-based, interactive key to all 179 species of Geophilomorpha (Chilopoda) recorded from Europe, including species of uncertain identity and those whose morphology is known partially only. The key is intended to assist in identification of subadult and adult specimens, by means of microscopy and simple dissection techniques whenever necessary. The key is freely available through the web at: http://www.biologia.unipd.it/chilokey/ and at http://www.interactive-keys.eu/chilokey/.

## General description

**Purpose:** At present, species identification of centipedes (Chilopoda) can be hardly carried on through effective identification tools, because adequate keys or diagnostic tables are available only for selected faunas and taxonomic subgroups. More often, the identification of specimens still requires retrieving and interpreting the original species descriptions, which are scattered in the primary taxonomic literature. Additionally, it often requires comparing the specimens of unknown identity with reference specimens that have been already identified by expert taxonomists.

This is true in particular for the diverse centipede order Geophilomorpha, which comprises 40% of all known species of Chilopoda (Bonato et al. 2011). This applies also to the European taxa, despite the fact that Europe has been investigated more thoroughly than all other continents. Indeed, modern and effective keys are available for different countries in northern and central Europe (e.g., Barber 2008 for Great Britain, Andersson et al. 2005 for Sweden), but these keys cover relatively poor faunas, whereas most of the European taxonomic diversity is harboured in the southern countries (Bonato and Minelli 2009). As for southern Europe, modern keys have been published only for very few areas (e.g., Stoev 2002 for Bulgaria, Iorio 2006 for part of France). At present, as a matter of fact, the identification of specimens from most part of Europe needs complementing outdated faunas and keys (above all, Brolemann 1930 for France, Matic 1972 for Romania) with taxonomic descriptions published in different languages, most often in regional taxonomic journals.

Recently, after completing a comprehensive synopsis of the European species of Geophilomorpha (Bonato and Minelli 2014), the rapid improvement of technologies and expertise in developing identification tools (Dallwitz 2000, Delgado Calvo-Flores et al. 2006, Penev et al. 2009, Cerretti et al. 2012) prompted us to build a matrix-based interactive tool to assist in species identification of geophilomorphs from the entire Europe, based on examination of morphological characters. This paper is intended as a “Data Paper” describing this tool, following Penev et al. (2009, 2012).

## Project details

**Project title:** ChiloKey.

**Personnel:** The authors.

**Figure 1.**
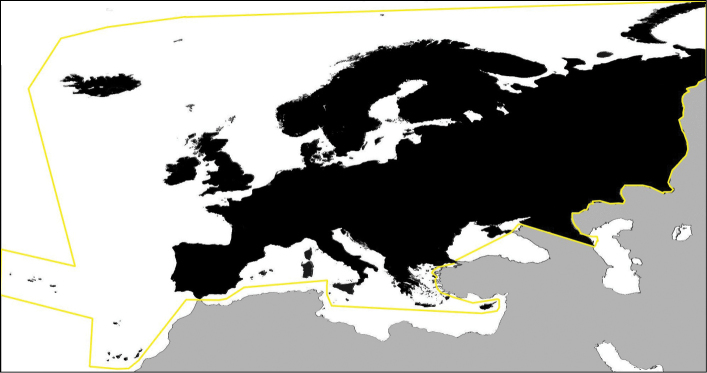
Conventional boundaries of the geographical area where ChiloKey is applicable.

**Study area descriptions/descriptor:** The key includes all species of Chilopoda Geophilomorpha that have been recorded from Europe. The area is delimited according to the conventional boundaries adopted in Fauna Europaea (de Jong 2013; Fig. 1).

### Taxonomic coverage

**General taxonomic coverage description:** The key includes a total 179 species (supplementary file 1), following the taxonomy and nomenclature recently proposed by Bonato and Minelli (2014).

**Applicability and identification units:** The key is intended to allow species-level identification of adult or subadult specimens, i.e. specimens with visible gonopods. The key allows also distinguishing the sex. Within a single species, the male and female phenotypes are treated operationally as distinct identification units, because some of the characters employed (different in different species) are also sexually dimorphic, and because some characters are applicable and effective in distinguishing species only for the male but not for the female or vice versa.

**Morphological terminology:** The key follows the conventional morphological terminology defined and illustrated by Bonato et al. (2010). Relevant body parts are labelled accordingly in the photos illustrating the character states (see below).

**Operational methods:** Only a few characters can be evaluated properly by means of a stereomicroscope, with incident light and at low magnification. In most cases, it is recommended to examine the specimen by means of a microscope, in transmitted light and at higher magnification, after including the specimen in a non-permanent microscopic slide.

The effective evaluation of some characters requires one or both of the following operations:

– detaching head from trunk. Recommended protocol: keeping the specimen under a stereomicroscope, on soft ground, with the dorsal side upwards; piercing the dorsal membrane between head and forcipular tergite repeatedly by means of a scalpel-like pin;

– detaching the maxillary complex from the remaining part of the head. Recommended protocol: after detaching head from trunk, keeping the head under a stereomicroscope, on a soft ground, with the ventral side upwards; piercing the lateral membranes connecting the second maxillary coxosternite to the pleurites repeatedly by means of a scalpel-like pin.

Further practical instructions are given by Pereira (2000) and Foddai et al. (2002).

**Figure 2.**
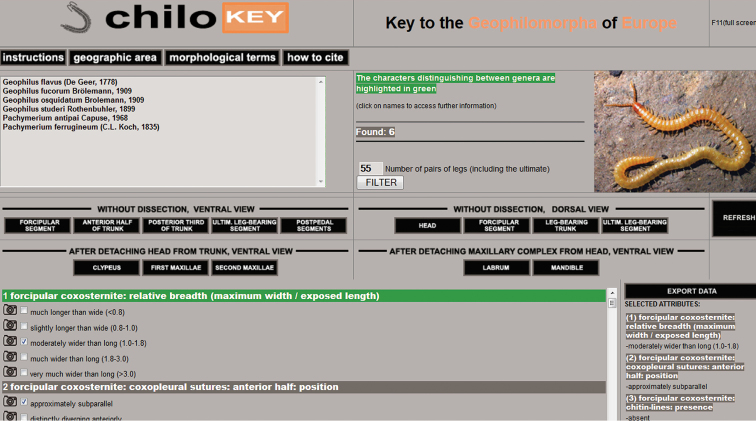
Screenshot of ChiloKey.

**Characters:** A total of 89 characters have been considered, including 51 binary characters, 37 multistate characters with 3–5 alternative states each, and a “filtering” character (number of leg-bearing segment), which is allowed to assume any integer value up to 999. Of the characters, 15 are depending on the state of another character, therefore their applicability is constrained. The characters have been selected giving priority to those with null or negligible intraspecific variation with respect to our state definitions, and to those that do not require dissection. Among the characters proposed and employed in the literature as diagnostic between species, we excluded those that have been found or suspected to be actually poorly effective, because of either intraspecific variability or artifactual origin (Bonato and Minelli 2014). In the key, characters are arranged in four sections according to the operations required or recommended for their evaluation (Fig. 2): (i) characters that can be evaluated on the entire body, keeping the specimen with the ventral side upwards (“without dissection, ventral view”; 54 characters); (ii) as before, but with the dorsal side upwards (“without dissection, dorsal view”; 13 characters); (iii) characters that can only be evaluated after detaching the head from the trunk, keeping both pieces with the ventral side upwards (“after detaching head from trunk, ventral view”; 12 characters); (iv) characters that can only be evaluated after removing the maxillary complex from the head and keeping both the maxillary complex and the remaining part of the head with their ventral side upwards (“after detaching maxillary complex from head, ventral view”; 9 characters). Within a section, characters are arranged in anatomical order, anterior to posterior, proximal to distal. Characters that are diagnostic at the genus level are highlighted. Most character states are illustrated by microscopic photos taken on specimens of representative species.

**Sources of data:** Characters have been coded for each species referring to selected published descriptions and illustrations (indicated in the species-file under “Main references”, see below; full references in supplementary file 2). Published information has been ignored when suspected or found to be inaccurate or based on misidentified specimens. For 67 species, data have been confirmed by us or integrated by direct examination of representative specimens, in the Bonato-Minelli collection, at the Department of Biology, University of Padova (supplementary file 1).

For a single species, more than one state have been assigned to a character whenever (i) there is interindividual variation, including sexual dimorphism, or (ii) the actual condition of the character is uncertain in the species, or (iii) subjectivity in evaluation could bring different users to score different states as present in the specimens at hand.

As to the number of leg-bearing segments, almost all species reported from Europe are known or expected to have interindividual variation in the number, the range of variation differing in different species, and the width of variation correlating approximately with the average value (Minelli et al. 2010). For a single species, when the number has been counted in more than a hundred specimens from at least two dozens of localities, the exact range between the recorded minimum and maximum has been adopted as a confident estimate of the intraspecific range of variation. Conversely, when the number has been counted in fewer specimens, and thus the variation in the species is probably underestimated, the intraspecific range has been estimated tentatively by considering the known average figure in the species and hypothesizing a 30% coefficient of variation (which is the average variation estimated in the intensely sampled species, as listed in supplementary file 1). This should not be taken as meaning that all segment numbers between the lowest and the highest value attributed to a given species have been actually recorded, or even occur in nature; discontinuities in the distribution of segment numbers within a species are well known, especially in species with very high segment number (e.g., Minelli and Bortoletto 1988).

**Species-files:** For any species, the following information is provided in a standardized way: “Valid name” of the species; “Family”; “Other names” (other names frequently used for the species after 1950, including synonyms, different generic combinations and alternative spellings); “Short description”; “Similar species” (selected species that could be misidentified, with main differential characters listed in parentheses); “Distribution” (known distribution in Europe, listing the main geographical areas from where the species has been reported reliably); “Taxonomic notes”; “Main references” (published sources that we considered for the morphology of the species; full citations in supplementary file 2).

## Taxonomic ranks

**Phylum:** Arthropoda

**Class:** Chilopoda

**Order:** Geophilomorpha

**Family:** Dignathodontidae, Geophilidae, Himantariidae, Linotaeniidae, Mecistocephalidae, Oryidae, Schendylidae

**Genus:** *Acanthogeophilus*, *Algerophilus*, *Arctogeophilus*, *Arenophilus*, *Bebekium*, *Bothriogaster*, *Clinopodes*, *Dicellophilus*, *Dignathodon*, *Diphyonyx*, *Escaryus*, *Espagnella*, *Eurygeophilus*, *Folkmanovius*, *Galliophilus*, *Geophilus*, *Gnathoribautia*, *Haplophilus*, *Haploschendyla*, *Henia*, *Himantariella*, *Himantarium*, *Nannophilus*, *Nothogeophilus*, *Nyctunguis*, *Orya*, *Pachymerium*, *Photophilus*, *Pleurogeophilus*, *Schendyla*, *Schizotaenia*, *Stenotaenia*, *Stigmatogaster*, *Strigamia*, *Thracophilus*, *Tuoba*

## Spatial coverage

**General spatial coverage:** The key includes all species of Chilopoda Geophilomorpha that have been recorded from Europe. In addition to the species whose validity is not questioned at present, included are also the nominal species whose taxonomic distinction is still uncertain and/or whose morphology is known only incompletely. Instead, we excluded alien species that have been occasionally reported from Europe but are probably not established in the wild, and also those that have been reported from Europe only by mistake.

**Coordinates:** between 33°50'24"N and 72°8'24"N Latitude; between 12°6'36"W and 43°45'0"E Longitude

**Natural collections description**

**Parent collection identifier:** Not applicable

**Collection name:** Department of Biology, University of Padova

**Collection identifier:** http://www.bio.unipd.it/

**Specimen preservation method:** Alcohol

## Methods

### Distribution

**a)** http://www.biologia.unipd.it/chilokey/

**b)** http://www.interactive-keys.eu/chilokey/

**Repository:** Department of Biology, University of Padova, Padova, Italy

**Metadata language:** English

**Date of metadata creation:** 2014-06-05

**Platform:** Windows Server 2003 - Microsoft Framework. NET 4

**Programming Language:** Asp.NET

**Data base:** MS Windows SQLSERVER, IIS6, NET 4.0

**Application version:** ChiloKey 1.0

**Update police:** The application can be augmented/updated only by, or in agreement with, the corresponding authors of this paper. Authors keep updated both the web application, by implementing new functions, and the data matrix, by improving encoded descriptions of terminal taxa. Every change can be monitored on the homepage and reported in the TXT export data file, by updating the number of the application version and by changing the date of the last modification to the data matrix. A short message on the homepage may describe differences from the previous version, if needed.

**Use of the primary data:** Primary data are available from the corresponding authors by agreement.

**Licence for the use of the key:** Creative Commons Attribution License 3.0 (CC-BY), which permits unrestricted use, distribution, and reproduction in any medium, provided the original authors and source are credited.

## Supplementary materials

Supplementary material 1 Species of Geophilomorpha included in ChiloKey 1.0 Data type: Species data. Species of Geophilomorpha included in ChiloKey 1.0, as released in 2014, and other species of Geophilomorpha reported from Europe but not included in ChiloKey 1.0, because either not established in the wild or most probably recorded only erroneously. This dataset is made available under the Open Database License (http://opendatacommons.org/licenses/odbl/1.0/). The Open Database License (ODbL) is a license agreement intended to allow users to freely share, modify, and use this Dataset while maintaining this same freedom for others, provided that the original source and author(s) are credited.Lucio Bonato, Alessandro Minelli, Massimo Lopresti, Pierfilippo Cerretti

Supplementary material 2 Bibliographic sources for the morphology matrix Data type: References list. Bibliographic sources for the morphology matrix of the species of Geophilomorpha in ChiloKey 1.0, as released in 2014. This dataset is made available under the Open Database License (http://opendatacommons.org/licenses/odbl/1.0/). The Open Database License (ODbL) is a license agreement intended to allow users to freely share, modify, and use this Dataset while maintaining this same freedom for others, provided that the original source and author(s) are credited.Lucio Bonato, Alessandro Minelli, Massimo Lopresti, Pierfilippo Cerretti
