# Supplementary material for: ChiloKey, an interactive identification tool for the geophilomorph centipedes of Europe (Chilopoda, Geophilomorpha)
Source: Zookeys. 2014 Sep 29;(443):1–9. doi: 10.3897/zookeys.443.7530 (PMC4205500; doi:10.3897/zookeys.443.7530)
Supplement: Supplementary material 1 — Species of Geophilomorpha included in ChiloKey 1.0 [file zookeys-443-001-s001.pdf]

ChiloKey, an interactive identification tool for the geophilomorph centipedes of Europe (Chilopoda, Geophilomorpha)

Lucio Bonato, Alessandro Minelli, Massimo Lopresti, Pierfilippo Cerretti

supplementary file 1

Species of Geophilomorpha included in ChiloKey 1.0, as released in 2014. Species are listed in alphabetic order by family, genus and species name.

species	sources	uncertain identity	direct examination of specimens	species considered to estimate number of leg-bearing segments
**Dignathodontidae**				
Dignathodon gracilis (Attems, 1952)	Attems 1952	x		
Dignathodon microcephalus (Lucas, 1846)	Latzel 1880; Sseliwanoff 1884; Berlese 1903; Brolemann 1930; Machado 1952; Verhoeff 1943a (also sub *Dignathodon clavigerum*), 1943b (also sub *D. microcephalum pachypus*), 1951 (sub *D. microcephalum sugens*); Matic 1972; Salinas 1990		x	x
Henia athenarum Pocock, 1891	Pocock 1891; Attems 1902 (sub *Henia biconica* and *H. Idomenei*), 1903 (sub *H. biconica*), 1929 (also sub *H. biconica* and *H. idomenei*); Minelli 1982a			
Henia attemsii Verhoeff, 1928	Verhoeff 1928; Attems 1929	x		
Henia bicarinata (Meinert, 1870)	Meinert 1870; Latzel 1880; Brolemann 1900; Berlese 1903; Verhoeff 1928, 1931 (both sub *Henia cassinensis*), 1938 (sub *H. bicarinata lapadensis*), 1943b (also sub *H. cassinensis*); Attems 1929; Brolemann 1930; Machado 1952; Matic 1972		x	
Henia brevis (Silvestri, 1896)	Silvestri 1896a; Verhoeff 1898 (sub *Chaetechelyne montana oblongocribellata*); Brolemann 1930 (sub *C. montana oblongocribellata*); Minelli 1982a; Barber 2009		x	x
Henia crinita Attems, 1902	Attems 1902, 1903, 1929	x		
Henia devia C.L. Koch, 1847	Koch 1963, 1867 (sub *Henia minor*); Verhoeff 1901 (sub *Scotophilus graecus*); Attems 1902, 1903 (sub *H. minor*), 1929 (also sub *H. minor*); Minelli 1982a		x	
Henia duboscqui (Verhoeff, 1943)	Verhoeff 1943a	x		
Henia flavescens (Attems, 1927)	Attems 1927, 1929	x		
Henia hirsuta Verhoeff, 1928	Verhoeff 1928; Attems 1929	x		
Henia illyrica (Meinert, 1870)	Meinert 1870; Latzel 1880; Berlese 1903; Folkmanovà 1928; Attems 1929; Verhoeff 1934 (sub *Henia illyrica oblonga*), 1938 (sub *Chaetechelyne herzegowinensis*); Matic 1972; Kaczmarek 1979		x	
Henia montana (Meinert, 1870)	Meinert 1870; Latzel 1880; Verhoeff 1928 (sub *Chaetechelyne vesuviana pharyngealis*); Brolemann 1930; Verhoeff 1951 (sub *C. pharyngealis*); Minelli 1982a (also sub *Henia pharyngealis*)		x	
Henia porosa (Verhoeff, 1941)	Verhoeff 1941, 1945; Ribarov 1987 (sub *Henia angelovi*)			
Henia pulchella (Meinert, 1870)	Meinert 1870; Attems 1902, 1929; Verhoeff 1928	x		
Henia ruffoi (Matic & Dărăbanţu, 1968)	Matic & Dărăbanţu 1968	x		
Henia taurica (Sseliwanoff, 1884)	Sseliwanoff 1884	x		

Henia valida (Attems, 1927)	Attems 1927, 1929; Matic & Dărăbanţu 1968 (sub *Chaetechelyne osellai*)	x		
Henia vesuviana (Newport, 1845)	Latzel 1880; Berlese 1903; Attems 1929; Machado 1952; Brolemann 1930; Verhoeff 1935a (sub *Chaetechelyne vesuviana helvetica*), 1943a (sub *C. corsica*), 1943b (sub *C. vittata*), 1843c (sub *C. grisea*), 1951 (sub *C. vesuviana helvetica*); Matic 1972; Barace & Herrera 1980; Barber 2009		x	x
**Geophilidae**				
Acanthogeophilus dentifer Minelli, 1982	Minelli 1982b			
Algerophilus hispanicus (Meinert, 1870)	Meinert 1870; Brolemann 1925; Machado 1953 (sub *Nesogeophilus mateui*); Bonato et al. 2012		x	
Arctogeophilus attemsi Folkmanová, 1956	Folkmanová 1956; Folkmanova & Dobroruka 1960	x		
Arctogeophilus inopinatus (Ribaut, 1911)	Ribaut 1911; Brolemann 1930; Demange 1981; Lewis & Kime 1988			
Arctogeophilus macrocephalus Folkmanová & Dobroruka, 1960	Folkmanová & Dobroruka 1960	x		
Arenophilus peregrinus Jones, 1989	Jones 1989; Gregory 1999; Barber 2009		x	
Bebekium mirabile Verhoeff, 1941	Verhoeff 1941, 1945	x		
Clinopodes carinthiacus (Latzel, 1880)	Latzel 1880; Verhoeff 1898 (sub *Geophilus flavidus trebevicensis*), 1934 (sub *G. trebevicensis*); Kaczmarek 1972 (sub *G. balcanicus*); Matic 1972 (sub *Clinopodes trebevicensis*); Bonato et al. 2011		x	x
Clinopodes escherichii (Verhoeff, 1896)	Verhoeff 1896, 1945; Attems 1901; Matic 1972; Bonato et al. 2011		x	x
Clinopodes flavidus C.L. Koch, 1847	Latzel 1880; Berlese 1903; Attems 1929; Verhoeff 1934; Matic 1972 (also sub *Clinopodes polytrichus*); Kaczmarek 1979; Bonato et al. 2011		x	x
Clinopodes intermedius Dărăbanţu & Matic, 1969	Dărăbanţu & Matic 1969; Matic 1972; Bonato et al. 2011	x		
Clinopodes rodnaensis (Verhoeff, 1938)	Verhoeff 1938; Matic 1972; Bonato et al. 2011		x	
Clinopodes skopljensis (Verhoeff, 1938)	Verhoeff 1938; Bonato et al. 2011	x		
Clinopodes verhoeffi Bonato, Iorio & Minelli, 2011	Verhoeff 1934 (sub *Geophilus flavidus porosus*); Matic 1972 (sub *Clinopodes porosus*); Bonato et al. 2011	x		
Clinopodes vesubiensis Bonato, Iorio & Minelli, 2011	Bonato et al. 2011		x	
Diphyonyx conjungens (Verhoeff, 1898)	Verhoeff 1898; Attems 1929; Bonato et al. 2008		x	
Diphyonyx sukacevi (Folkmanová, 1956)	Folkmanová 1956; Bonato et al. 2008	x		
Eurygeophilus multistiliger (Verhoeff, 1899)	Machado 1952; Bonato et al. 2006		x	
Eurygeophilus pinguis (Brölemann, 1898)	Bonato et al. 2006; Barber 2009		x	
Folkmanovius paralellus Dobroruka, 1957	Dobroruka 1957	x		
Galliophilus beatensis Ribaut & Brolemann, 1927	Brolemann 1927, 1930			
Geophilus aenariensis Verhoeff, 1942	Verhoeff 1942, 1943a, 1943c	x		
Geophilus aetnensis Verhoeff, 1928	Verhoeff 1928, 1943a (sub *Geophilus evisensis*); Brolemann 1930 (sub *G. insculptus debilis*); Manfredi 1956 (sub *G. henroti*), 1957 (sub *G. aetnensis pollinensis*)	x		
Geophilus algarum Brölemann, 1909	Brölemann 1909, 1930 (also sub *Geophilus algarum* var. *decipiens*); Demange 1961 (sub *G. algarum* var. *decipiens*); Lewis 1962			
Geophilus alpinus Meinert, 1870	Meinert 1870; Attems 1895, 1929 (both sub *Geophilus insculptus*); Verhoeff 1895 (sub *G. insculptus*); Brolemann 1930 (sub *G. insculptus*); Matic 1972 (sub *G. insculptus*); Christian 1996 (sub *G. insculptus*); Barber 1999, 2009 (sub *G. insculptus*); Barber & Jones 1999 (sub *G. insculptus*); Andersson et al. 2005 (sub *G. insculptus*)		x	x
Geophilus alzonis Attems, 1952	Attems 1952	x		
Geophilus bluncki Verhoeff, 1928	Verhoeff 1928	x		

Geophilus bobolianus Verhoeff, 1928	Verhoeff 1928, 1930, 1934 (also sub *Geophilus longicornis aternanus*), 1942 (sub *G. longicornis bobolianus serratulus*), 1943c (also sub *G. taorminensis aternanus*)	x		
Geophilus bosniensis Verhoeff, 1895	Verhoeff 1895; Verhoeff 1896; Verhoeff 1898	x		
Geophilus carpophagus Leach, 1815	Attems 1929; Brölemann 1930; Machado 1952; Eason 1964; Matic 1972; Kaczmarek 1979; Barace & Herrera 1980; Arthur et al. 2001; Andersson et al. 2005; Barber 2009; Gregory & Barber 2010; Bonato & Minelli 2011		x	x
Geophilus chalandei Brölemann, 1909	Brölemann 1909, 1930; Barace & Herrera 1980			
Geophilus easoni Arthur, Foddai, Kettle, Lewis, Luczynski & Minelli, 2001	Arthur et al. 2001; Haswell et al. 2006; Barber 2009; Bonato & Minelli 2011		x	
Geophilus electricus (Linnaeus, 1758)	Latzel 1880; Attems 1903, 1929; Berlese 1903; Brolemann 1930; Eason 1964; Matic 1972; Misioch 1978; Kaczmarek 1979; Andersson et al. 2005; Barber 2009		x	x
Geophilus flavus (De Geer, 1778)	Berlese 1903 (sub *Geophilus longicornis*); Attems 1928 (sub *Pachymerium tristanicum*), 1929 (sub *G. longicornis*); Brolemann 1930 (sub *Necrophloeophagus longicornis*); Eason 1964 (sub *N. longicornis*); Matic 1972 (sub *N. longicornis*); Misioch 1978 (sub *N. longicornis*); Christian 1996; Andersson et al. 2005; Barber 2009; Simaiakis et al. 2010		x	x
Geophilus fossularum Verhoeff, 1943	Verhoeff 1943a	x		
Geophilus frigidanus Verhoeff, 1928	Verhoeff 1928	x		
Geophilus fucorum Brölemann, 1909	Brölemann 1909, 1930; Verhoeff 1928 (sub *Geophilus longicornis taorminensis*), 1931 (sub *G. ruinarum*), 1942 (sub *G. longicornis taorminensis*), 1943c (sub *G. taorminensis*); Iorio 2006		x	
Geophilus gavoyi Chalande, 1910	Chalande & Ribaut 1910; Attems 1929; Brolemann 1930; García Ruiz & Serra 2000			
Geophilus guanophilus Verhoeff, 1939	Verhoeff 1939	x		
Geophilus ibericus (Attems, 1952)	Attems 1952	x		
Geophilus joyeuxi Léger & Duboscq, 1903	Léger & Duboscq 1903; Brolemann 1930			
Geophilus labrofissus Verhoeff, 1938	Verhoeff 1938	x		
Geophilus madeirae Latzel, 1895	Latzel 1895	x		
Geophilus minimus Verhoeff, 1928	Verhoeff 1928; Minelli 1983a; Foddai & Minelli 1999		x	
Geophilus nanus Attems, 1952	Attems 1952	x		
Geophilus nesiotes Attems, 1903	Attems 1903, 1929	x		
Geophilus oligopus (Attems, 1895)	Attems 1895, 1927 (sub *Geophilus pauropus*), 1929 (also sub *G. pauropus*); Verhoeff 1928 (sub *G. noricus*); Minelli 1983a; Christian 1996; Barber 1999; Foddai & Minelli 1999; Dányi 2007		x	
Geophilus orae Verhoeff, 1943	Verhoeff 1943c	x		
Geophilus osquidatum Brölemann, 1909	Brolemann 1909, 1930; Verhoeff 1928 (sub *Geophilius scillyensis*); Turk 1947 (sub *G. scillyensis*); Blower 1961; Eason 1964; Salinas 1990; Barber 2009		x	
Geophilus pauciporus (Machado, 1952)	Machado 1952	x		
Geophilus pellekanus Attems, 1903	Attems 1903, 1929	x		
Geophilus persephones Foddai & Minelli, 1999	Foddai & Minelli 1999			
Geophilus piae Minelli, 1983	Minelli 1983a; Foddai & Minelli 1999		x	
Geophilus pinivagus Verhoeff, 1928	Verhoeff 1928	x		
Geophilus promontorii Verhoeff, 1928	Verhoeff 1928; Matic 1972	x		

Geophilus proximus C.L. Koch, 1847	Latzel 1880; Attems 1901 (sub *Geophilus ganonotus*), 1929 (also sub *G. commutatus* and *G. ganonotus*); Chalande 1909; Brolemann 1930; Hammer 1931; Lignau 1933 (sub *G. eremophilus*); Jawlowski 1949; Palmén 1949; Demange 1959; Enghoff 1971; Matic 1972; Kaczmarek 1979; Zalesskaja et al. 1982; Barber & Jones 1999; Andersson et al. 2005; Bonato et al. 2005; Barber 2009		x	x
Geophilus punicus Silvestri, 1896	Silvestri 1896b, 1898a, 1898b	x		
Geophilus pusillifrater Verhoeff, 1898	Verhoeff 1898; Lewis 1961; Eason 1964; Iorio 2006; Barber 2009			
Geophilus pygmaeus Latzel, 1880	Latzel 1880; Verhoeff 1895, 1896, 1898, 1934 (sub *Geophilus larii*), 1938, 1940; Silvestri 1896a (sub *G. cispadanus*); Attems 1929		x	
Geophilus pyrenaicus Chalande, 1909	Brölemann 1908, 1930; Chalande 1909; Salinas 1990			
Geophilus ribauti Brölemann, 1908	Brölemann 1908, 1909, 1930; Kime & Iorio 2010			
Geophilus richardi Brölemann, 1904	Brölemann 1904, 1909, 1930; Foddai & Minelli 1999		x	
Geophilus seurati Brolemann, 1924	Meinert 1870 (sub *Geophilus gracilis*); Brolemann 1924; Attems 1929 (sub *G. gracilis*); Barber 2009 (sub *G. gracilis*); Iorio 2006 (sub *G. gracilis*)		x	
Geophilus silesiacus Haase, 1881	Haase 1881; Kaczmarek 1979	x		
Geophilus strictus Latzel, 1880	Latzel 1880; Verhoeff 1895, 1896, 1898; Attems 1929; Stoev 2002	x		
Geophilus studeri Rothenbühler, 1899	Rothenbühler 1899; Verhoeff 1901, 1928, 1937a (also sub *Geophilus silvaenigrae*), 1939 (sub *G. silvaenigrae* and *G. padbergi*); Faës 1902; Attems 1929; Misioch 1979 (sub *G. silvaenigrae*); Spelda 1991			
Geophilus tenellus L. Koch, 1882	Koch 1882	x		
Geophilus truncorum Bergsøe & Meinert, 1866	Bergsøe & Meinert 1866; Attems 1929; Brolemann 1930; Machado 1952; Eason 1964; Misioch 1978; Kaczmarek 1979; Barace & Herrera 1980; Andersson et al. 2005; Barber 2009		x	x
Geophilus ungviculatus Daday, 1889	Daday 1889b; Attems 1929	x		
Geophilus vinciguerrae Silvestri, 1895	Silvestri 1895; Minelli 1983b	x		
Gnathoribautia bonensis (Meinert, 1870)	Newport 1844 (sub *Necrophloeophagus punctiventris*); Meinert 1870; Porat 1871 (sub *Geophilus hirsutus*); Verhoeff 1896 (sub *Mecistocephalus lusitanus*); Attems 1900 (sub *M. agricola*); Berlese 1903; Machado 1952		x	
Gnathoribautia syriaca (Attems, 1903)	Attems 1903, 1929	x		
Nothogeophilus turki Lewis, Jones & Keay, 1988	Lewis et al. 1988; Barber 2009			
Pachymerium antipai Capuse, 1968	Capuse 1968; Matic 1972	x		
Pachymerium atticum Verhoeff, 1901	Verhoeff 1901; Capuse 1968; Matic 1972	x		
Pachymerium coiffaiti Demange, 1959	Demange 1959 (also sub *Pachymerium ferrugineum maderianum*)	x		
Pachymerium ferrugineum (C.L. Koch, 1835)	Latzel 1880; Attems 1903 (sub *P. caucasicus*), 1929; Berlese 1903; Machado 1952; Brolemann 1900, 1930; Verhoeff 1934; Eason 1964; Capuse 1968; Matic 1972; Salinas 1990; Andersson et al. 2005; Barber 2009; Simaiakis et al. 2010		x	x
Pachymerium minutum (Sseliwanoff, 1884)	Sseliwanoff 1884	x		
Pachymerium tyrrhenum Verhoeff, 1934	Verhoeff 1934	x		
Photophilus griseus Folkmanová, 1928	Folkmanová 1928, 1929	x		
Pleurogeophilus herzegowinensis (Verhoeff, 1901)	Verhoeff 1901; Attems 1959	x		
Pleurogeophilus mediterraneus (Meinert, 1870)	Latzel 1880; Attems 1903; Verhoeff 1928 (sub *Pleurogeophilus mediterraneus glandulosus*); Brolemann 1930		x	x
Pleurogeophilus vetustus Silvestri, 1907	Silvestri 1907	x		
Schizotaenia sp.			x	
Stenotaenia antecribellata (Verhoeff, 1898)	Verhoeff 1898; Attems 1903, 1929; Bonato & Minelli 2008	x	x	

Stenotaenia cribelliger (Verhoeff, 1898)	Verhoeff 1898; Attems 1929; Bonato & Minelli 2008	x		
Stenotaenia linearis (C.L. Koch, 1835)	Latzel 1880; Berlese 1903; Attems 1929; Brolemann 1930; Eason 1964; Matic 1972; Keay 1994; Andersson et al. 2005; Barber 2009; Bonato & Minelli 2008		x	x
Stenotaenia naxia (Verhoeff, 1901)	Verhoeff 1901, 1902 (sub *Geophilus graecus*), 1925; Bonato & Minelli 2008	x	x	
Stenotaenia palpiger Attems, 1903	Attems 1903, 1929; Bonato & Minelli 2008	x		
Stenotaenia rhodopensis (Kaczmarek, 1970)	Kaczmarek 1970; Bonato & Minelli 2008	x		
Stenotaenia romana (Silvestri, 1895)	Silvestri 1895; Verhoeff 1928 (sub *Geophilus silvestrii*); Minelli 1983a; Bonato & Minelli 2008		x	x
Stenotaenia sorrentina (Attems, 1903)	Attems 1903; Verhoeff 1925 (sub *Geophilus linearis abbreviatus*); Matic 1972 (sub *Clinopodes abbreviatus*); Lewis 1994 (sub *G. linearis*); Bonato & Minelli 2008		x	x
Stenotaenia sturanyi (Attems, 1903)	Attems 1903; Attems 1929; Bonato & Minelli 2008		x	
Tuoba poseidonis (Verhoeff, 1901)	Verhoeff 1901; Berlese 1903; Attems 1929; Brolemann 1930; Verhoeff 1943a (also sub *Geophilus poseidonis siscensis*); Lewis 1963; Andersson et al. 2005		x	
Tuoba zograffi (Brölemann, 1900)	Brölemann 1900	x		
**Himantariidae**				
Bothriogaster signata (Kessler, 1874)	Verhoeff 1901 (sub *Bothriogaster* sp. pl.), 1908 (sub *Bothriogaster cyrenaica*), 1925 (sub *Bothriogaster* sp. pl.); Chalande & Ribaut 1909; Attems 1910 (sub *B. signata megalocycla*), 1926, 1929 (both sub *Bothriogaster* sp. pl.); Lewis 1986 (sub *B. aegyptiaca*)		x	x
Haplophilus arcisherculis (Brölemann, 1904)	Brolemann 1904, 1930; Verhoeff 1938 (sub *Stigmatogaster simrothi*); Vadell & Pons 2009		x	
Haplophilus dimidiatus (Meinert, 1870)	Chalande & Ribaut 1909; Attems 1929; Brolemann 1900, 1930; Machado 1952; Salinas 1990		x	x
Haplophilus excavatus (Verhoeff, 1924)	Verhoeff 1924; Brolemann 1930; Matic & Dărăbanţu 1969 (sub *Nesoporogaster hispanica*)			
Haplophilus neglectus Brolemann, 1926	Brolemann 1926, 1930; Attems 1929; Barber 2009	x		
Haplophilus sardous Verhoeff, 1901	Verhoeff 1901; Manfredi 1956 (sub *Stigmatogaster sardoa dorgalina*); Minelli 1983a	x		
Haplophilus souletinus (Brolemann, 1907)	Chalande & Ribaut 1909; Brolemann 1930; Eason 1962, 1964; Matic & Dărăbanţu 1969 (*Nesoporogaster mediterranea*); Barber 2009		x	x
Haplophilus subterraneus (Shaw, 1794)	Meinert 1870; Chalande & Ribaut 1909; Attems 1929; Brolemann 1930; Eason 1964; Lesniewska & Wojciechowski 1992; Andersson et al. 2005; Barber 2009; Lesniewska 2012		x	x
Haplophilus superbus (Meinert, 1870)	Meinert 1870 (also sub *Himantarium filum*); Chalande & Ribaut 1909; Attems 1929; Brolemann 1932, 1947; Verhoeff 1951			
Himantariella balearica Capuse, 1975	Capuse 1975			
Himantariella scutellaris Brolemann, 1926	Brolemann 1926, 1930			
Himantarium europaeum (Chalande & Ribaut, 1909)	Chalande & Ribaut 1909; Attems 1929; Brolemann 1930		x	
Himantarium gabrielis (Linnaeus, 1767)	Meinert 1870; Latzel 1880; Berlese 1903 (also sub *Himantarium rugulosum*); Chalande & Ribaut 1909 (sub *H. rugulosum*); Attems 1929; Brolemann 1930; Matic 1972; Simaiakis 2009		x	x
Stigmatogaster gracilis (Meinert, 1870)	Latzel 1880; Berlese 1903; Chalande & Ribaut 1909; Ribaut 1910; Attems 1929; Brolemann 1930; Verhoeff 1934		x	x
Thracophilus bulgaricus Verhoeff, 1926	Verhoeff 1926; Attems 1929; Kaczmarek 1969a; Matic & Dărăbanţu 1974 (also sub *Thracophilus beroni*)			
Thracophilus chiosensis Stavropoulos & Matic, 1990	Stavropoulos & Matic 1990	x		
Thracophilus cilicius Attems, 1947	Attems 1947; Simaiakis et al. 2013	x		
Thracophilus subterraneus Verhoeff, 1943	Verhoeff 1943d; Attems 1947, 1951 (both sub *Thracophilus monoporus*), 1959 (sub *T. bulgaricus* and *T. monoporus*)			

**Linotaeniidae**				
Strigamia acuminata (Leach, 1815)	Brolemann 1930; Verhoeff 1935b; Eason 1964; Matic 1972; Koren 1986; Iorio 2004, 2005; Andersson et al. 2005; Barber 2008, 2009; Bonato et al. 2012		x	x
Strigamia cottiana (Verhoeff, 1935)	Verhoeff 1935b (also sub *Scolioplanes dalmazzensis*); Iorio 2008; Bonato et al. 2012		x	
Strigamia crassipes (C.L. Koch, 1835)	Verhoeff 1928, 1935b; Brolemann 1930 (sub *Scolioplanes acuminatus* partim); Machado 1952; Eason 1964; Matic 1972; Koren 1986; Iorio 2004, 2005; Andersson et al. 2005; Barber 2008, 2009; Bonato et al. 2012		x	x
Strigamia crinita (Attems, 1929)	Attems 1929; Verhoeff 1935b; Matic & Dărăbanţu 1968; Matic 1972	x		
Strigamia engadina (Verhoeff, 1935)	Verhoeff 1935b (also sub *Scolioplanes mendelanus* and *S. engadinus rodnaensis*); Stoev 2002; Bonato et al. 2012	x		
Strigamia herzegowinensis (Verhoeff, 1935)	Verhoeff 1935b; Dobroruka 1977	x		
Strigamia lutea Matic, 1985	Matic 1985	x		
Strigamia maritima (Leach, 1817)	Brolemann 1930; Eason 1964; Andersson et al. 2005; Barber 2008, 2009; Horneland & Meidell 2009; Bonato et al. 2012		x	x
Strigamia olympica Dobroruka, 1977	Dobroruka 1977	x		
Strigamia paucipora Matic, 1985	Matic 1985	x		
Strigamia pusilla (Sseliwanoff, 1884)	Sseliwanoff 1884; Verhoeff 1935b (sub *Scolioplanes perkeo*); Loksa 1962 (sub *S. pseudopusillus*); Kaczmarek 1981 (sub *Strigamia perkeo*); Zalesskaja et al. 1982; Dányi 2006; Bonato et al. 2012		x	
Strigamia transsilvanica (Verhoeff, 1928)	Verhoeff 1928, 1935b; Matic 1972; Folkmanová 1952; Kaczmarek 1979; Koren 1986; Stoev 2002; Bonato et al. 2005, 2012; Iorio 2005		x	
**Mecistocephalidae**				
Dicellophilus carniolensis (C.L. Koch, 1847)	Latzel 1880; Attems 1929; Eason 1964; Matic 1972; Koren 1986; Mikos 1991; Bonato et al. 2010		x	
**Oryidae**				
Orya barbarica (Gervais, 1835)	Meinert 1870; Attems 1929; Turk 1955 (sub *Orya almohadensis*); Demange 1961 (sub *O. panousei*)		x	
**Schendylidae**				
Escaryus haasei (Sseliwanoff, 1884)	Sseliwanoff 1884	x		
Escaryus ornatus Folkmanová, 1956	Folkmanová 1956	x		
Escaryus retusidens Attems, 1904	Attems 1904, 1929; Folkmanová 1956; Titova 1972			
Espagnella franzi Attems, 1952	Attems 1952	x		
Haploschendyla grantii (Pocock, 1891)	Meinert 1870 (sub *Geophilus barbaricus*); Pocock 1891; Latzel 1895 (sub *G. barbaricus*); Attems 1903 (sub *Pectiniunguis europaeus*); Brolemann & Ribaut 1912 (sub *Haploschendyla bordei*); Demange 1959 (sub *H. europaea latzeli*)		x	
Haploschendyla splitensis (Verhoeff, 1938)	Verhoeff 1938	x		
Hydroschendyla submarina (Grube, 1872)	Berlese 1903; Brolemann & Ribaut 1912; Brolemann 1930; Eason 1964; Andersson et al. 2005; Barber 2009		x	
Nannophilus ariadnae Attems, 1902	Attems 1902; Brolemann & Ribaut 1912; Demange 1959	x		
Nannophilus eximius (Meinert, 1870)	Meinert 1870; Brolemann & Ribaut 1912; Attems 1929; Demange 1959		x	
Nannophilus melanostictus (Attems, 1911)	Attems 1911	x		
Nannophilus vandeli Demange, 1959	Demange 1959	x		
Nyctunguis persimilis Attems, 1932	Attems 1932; Christian 1996			
Schendyla apenninorum (Brölemann & Ribaut, 1911)	Brölemann & Ribaut 1912	x		
Schendyla armata Brölemann, 1901	Brölemann 1901, 1930; Brölemann & Ribaut 1912; Verhoeff 1934 (sub *Brachyschendyla armata broelemanni*)		x	

Schendyla aternana (Verhoeff, 1934)	Verhoeff 1934	x		
Schendyla capusei (Dărăbanţu & Matic, 1969)	Dărăbanţu & Matic 1969; Matic 1972	x		
Schendyla carniolensis Verhoeff, 1902	Verhoeff 1902, 1937b (sub *Schendyla nemorensis quarnerana*); Brölemann & Ribaut 1912 (sub *S. zonalis*); Brolemann 1930 (sub *S. zonalis*); Bagnall 1935 (sub *S. zonalis*); Eason 1964 (sub *S. zonalis*); Matic 1972 (sub *S. zonalis*); Barber 2009		x	x
Schendyla dalmatica Attems, 1904	Attems 1904, 1929	x		
Schendyla delicatula Kaczmarek, 1969	Kaczmarek 1969b	x		
Schendyla dentata (Brölemann & Ribaut, 1911)	Brölemann & Ribaut 1912; Brolemann 1930; Barber & Eason 1970; Barber & Jones 1999; Andersson et al. 2005; Barber 2009			
Schendyla gracillima Verhoeff, 1934	Verhoeff 1934	x		
Schendyla hispanica (Attems, 1952)	Attems 1952	x		
Schendyla mediterranea Silvestri, 1898	Brölemann & Ribaut 1912; Brolemann 1930; Dărăbanţu 1972; Matic 1972		x	
Schendyla monodi (Brolemann, 1924)	Brolemann 1924, 1930; Demange 1961; Verhoeff 1951 (sub *Brachyschendyla viridis*)			
Schendyla monoeci Brölemann, 1904	Brölemann 1904, 1930; Brölemann & Ribaut 1912; Eason 1964; Matic 1972; Barber 2009			
Schendyla negreai (Dărăbanţu & Matic, 1969)	Dărăbanţu & Matic 1969; Matic 1972	x		
Schendyla nemorensis (C.L. Koch, 1837)	Berlese 1903; Attems 1929; Brolemann 1930; Eason 1964; Matic 1972; Koren 1986; Kaczmarek 1980; Andersson et al. 2005; Barber 2009		x	x
Schendyla peyerimhoffi Brölemann & Ribaut, 1911	Brölemann & Ribaut 1912; Machado 1952; Lewis 1961; Eason 1964; Barber 2009			
Schendyla tyrolensis (Meinert, 1870)	Meinert 1870; Brolemann & Ribaut 1912 (sub *Brachyschendyla montana*); Brolemann 1927 (sub *B. montana prominens*), 1930 (sub *B. montana*); Attems 1929 (sub *B. montana*); Kaczmarek 1969b (sub *B. montana balcanica*); Matic 1972 (sub *B. montana*); Koren 1986 (sub *B. montana*)		x	x
Schendyla varnensis (Kaczmarek, 1969)	Kaczmarek 1969b; Matic 1972	x		
Schendyla verneri (Folkmanová & Dobroruka, 1960)	Folkmanová & Dobroruka 1960	x		
Schendyla vizzavonae Léger & Duboscq, 1903	Léger & Duboscq 1903; Brölemann & Ribaut 1912; Brolemann 1930; Verhoeff 1934 (sub *Schendyla pellicensis*), 1938, 1943a	x		
Schendyla walachica Verhoeff, 1900	Verhoeff 1900; Brölemann & Ribaut 1912; Kaczmarek 1969b (sub *Schendyla walachica rhodopensis*); Matic 1972			

Other species of Geophilomorpha reported from Europe but not included in ChiloKey 1.0, as released in 2014, because either not established in the wild or most probably recorded only erroneously. Species are listed in alphabetic order by family, genus and species name.

species	uncertain identity	not established in the wild	erroneous record
**Geophilidae**			
Arctogeophilus wolfi (Ribaut, 1911)			x
Geophilus arenarius Meinert, 1870			x
Geophilus attenuatus Say, 1821	x		x
Geophilus pusillus Meinert, 1870			x
Porethus pauciporus Chamberlin, 1952	x		x
Steneurytion sp.	x	x	
**Himantariidae**			
Himantarium mediterraneum Meinert, 1870			x
Himantarium tenue Latzel, 1886	x		x
**Mecistocephalidae**			
Mecistocephalus guildingii Newport, 1843		x	
Mecistocephalus maxillaris (Gervais, 1837)	x	x	
Mecistocephalus punctifrons Newport, 1843			x
Tygarrup javanicus Attems, 1929		x	
**Oryidae**			
Orphnaeus brevilabiatus (Newport, 1845)		x	
