# Supplementary material for: ChiloKey, an interactive identification tool for the geophilomorph centipedes of Europe (Chilopoda, Geophilomorpha)
Source: Zookeys. 2014 Sep 29;(443):1–9. doi: 10.3897/zookeys.443.7530 (PMC4205500; doi:10.3897/zookeys.443.7530)
Supplement: Supplementary material 2 — Bibliographic sources for the morphology matrix [file zookeys-443-001-s002.pdf]

ChiloKey, an interactive identification tool for the geophilomorph centipedes of Europe (Chilopoda, Geophilomorpha)

Lucio Bonato, Alessandro Minelli, Massimo Lopresti, Pierfilippo Cerretti

supplementary file 2

Bibliographic sources for the morphology matrix of the species of Geophilomorpha in ChiloKey 1.0, as released in 2014.

Andersson, G., Meidell, B., Scheller, U., Djursvoll, P., Gärdenfors, U., Budd, G., Bergström, J. & Xianguang, H. (2005) *Mångfotingar (Myriapods)*. Nationalnyckeln till Sveriges flora och fauna. Artdatabanken, SLU, Uppsala, 351 pp.

Arthur et al. (2001) Analysis of segment number and enzyme variation in a centipede reveals a cryptic species, *Geophilus easoni* sp. nov., and rises questions about speciation. *Biological Journal of the Linnean Society*, 74, 489-499.

Attems, C.G. (1895) Die Myriopoden Steiermarks. *Sitzungsberichte der kaiserlichen Akademie der Wissenschaften in Wien. Mathematisch-naturwissenschaftliche Klasse*, 104, 117-238.

Attems, C.G. (1900) Über die Färbung von *Glomeris* und Beschreibung neuer oder wenig gekannter paläarktischer Myriopoden. *Archiv für Naturgeschichte*, 66, 297-320.

Attems, C.G. (1901) Myriopoden. *In:* Horváth G. (Ed.), *Zoologische Ergebnisse der dritten asiatischen Forschungsreise des Grafen Eugen Zichy*, Victor Hornyánszky, Budapest, and Karl W. Hiersemann, Leipzig, pp. 275-310.

Attems, C.G. (1902) Myriopoden von Kreta, nebst Beiträgen zur allgemeinen Kenntnis einiger Gattungen. *Sitzungsberichte der kaiserlichen Akademie der Wissenschaften in Wien*. *Mathematisch-naturwissenschaftlichen Klasse*, 111 (1), 527-614.

Attems, C.G. (1903) Synopsis der Geophiliden. *Zoologische Jahrbücher. Abteilung für Systematik, Geographie und Biologie der Tiere*, 18, 155-302.

Attems, C.G. (1904) Neue palaearktische Myriopoden nebst Beiträge zur Kenntniss einiger alten Arten. *Archiv für Naturgeschichte*, 70, 179-196.

Attems, C.G. (1910) Ergebnisse der mit Subvention aus der Erbschaft Treitl unternommenen zoologischen Forschungsreise Dr. Franz Werner's nach dem aegyptischen Sudan und Nord-Uganda. XVI. Myriopoden. *Sitzungsberichte der kaiserlichen Akademie der Wissenschaften in Wien*. *Mathematisch-naturwissenschaftlichen Klasse*, 119: 355-360.

Attems, C.G. (1911) Description de myriopodes nouveaux recueillis par M. Henri Gadeau de Kerville pendant son voyage zoologique en Syrie. *Bulletin de la Société des Amis des Sciences naturelles de Rouen*, 46, 61-67.

Attems, C.G. (1926) Étude sur les myriapodes recueillis par M. Henri Gadeau de Kerville pendant son voyage zoologique en Syrie. *In:* V*oyage zoologique d'Henri Gadeau de Kerville en Syrie (Avril-Juin 1908)*. J.-B. Baillière et fils, Paris, vol. 1, pp. 221-264.

Attems, C.G. (1927) Myriopoden aus dem Nördlichen und Östlichen Spanien, gesammelt vor Dr. F. Haas in den Jahren 1914-1919. *Abhandlungen der Senckenbergischen Naturforschenden Gesellschaft*, 39, 235-289.

Attems, C.G. (1928) The Myriopoda of South Africa. *Annals of the South African Museum*, 26, 1-431.

Attems, C.G. (1929) Myriapoda I: Geophilomorpha. *Das Tierreich*, 52. De Gruyter, Berlin, 388 pp.

Attems, C.G. (1932) *Étude sur les Myriopodes recueillis par M. Henry Gadeau de Kerville pendant son voyage zoologique en Asie-Mineure (Avril-Mai 1912)*. Extrait du tome premier (en cours de rédaction) du Voyage zoologique d'Henri Gadeau de Kerville en Asie Mineure (Avril-Mai 1912). Lecerf, Rouen, 16 pp.

Attems, C.G. (1947) Neue Geophilomorpha des Wiener Museums. *Annalen des naturhistorischen Museums in Wien*, 55, 50-149.

Attems, C.G. (1951) Neue Höhlen-Myriopoden, gesammelt von Professor Absolon. *Anzeiger der Österreichischen Akademie der Wissenschaften. Mathematisch-naturwissenschaftliche Klasse*, 10, 253-257.

Attems, C.G. (1952) Myriopoden der Forschungsreise Dr. H. Franz in Spanien 1951 nebst über die gesamte Iberische Myriopodenfauna. *Eos, Revista Española de Entomología*, 28, 323-366.

Attems, C.G. (1959) Die Myriopoden der Höhlen der Balkanhalbinsel. Nach dem Material der "Biospeologica balcanica". *Annalen des naturhistorischen Museums in Wien*, 63, 281-406.

Bagnall, R.S. (1935) Notes on British chilopods (centipedes). I. *Annals and Magazine of natural History*, 15, 473-479.

Barace, J. & Herrera, L. (1980) Estudio faunistico del macizo de Quinto Real. III. Miriápodos quilópodos (Myriapoda Chilopoda). *Publicaciones de Biología de la Universidad de Navarra*, 4, 1-26.

Barber A.D. (1999) *Geophilus insculptus* or *Geophilus oligopus*? *Bulletin of the British Myriapod Group*, 15, 26-27.

Barber, A.D. (2008) Key to the identification of British centipedes. *Field Study Council Occasional Publications*, 130, 96 pp.

Barber, A.D. (2009) Centipedes. *Synopses of the British Fauna*, The Linnean Society of London, 58, 228 pp.

Barber, A.D. & Eason, E.H. (1970) On *Brachyschendyla dentata* Brölemann & Ribaut (Chilopoda, Schendylidae), a centipede new to Britain. *Journal of Natural History*, 4, 79-84.

Barber, A.D. & Jones, R.E. (1999) A description of *Geophilus proximus*. *Bulletin of the British Myriapod Group*, 15, 19-25.

Bergsøe, V. & Meinert, F. (1866) Danmarks Geophiler. *Naturhistorisk Tidsskrift*, 4, 81-103.

Berlese, A. (1903) *Myriapoda, Acari, Scorpiones hucusque in Italia reperta*. Padova, Tipografia del Seminario, pages not numbered.

Blower, J.G. (1961) On some new and little known British centipedes. *Annals and Magazine of natural History*, 4, 183-187.

Bonato, L., Barber, A. & Minelli, A. (2006) The European centipedes hitherto referred to *Eurygeophilus*, *Mesogeophilus* and *Chalandea* (Chilopoda, Geophilomorpha): taxonomy, distribution and geographic variation of the segment number. *Journal of Natural History*, 40, 415-438.

Bonato, L., Dányi, L. & Minelli, A. (2010) Morphology and phylogeny of *Dicellophilus*, a centipede genus with highly disjunct distribution (Chilopoda, Mecistocephalidae). *Zoological Journal of the Linnean Society*, 158, 501-532.

Bonato, L., Dányi, L., Socci, A.A. & Minelli, A. (2012) Species diversity of *Strigamia* (Chilopoda: Linotaeniidae): a preliminary synthesis. *Zootaxa*, 3593, 1-39.

Bonato, L., Iorio, E. & Minelli, A. (2011) The centipede genus *Clinopodes* C.L. Koch, 1847 (Chilopoda, Geophilomorpha, Geophilidae): reassessment of species diversity and distribution, with a new species from the Maritime Alps (France). *Zoosystema*, 33, 175-205.

Bonato, L. & Minelli, A. (2008) *Stenotaenia* Koch, 1847: a hitherto unrecognized lineage of western Palaearctic centipedes with unusual diversity in body size and segment number (Chilopoda: Geophilidae). *Zoological Journal of the Linnean Society*, 153, 253-286.

Bonato, L., Minelli, A. & Spungis, V. (2005) Geophilomorph centipedes of Latvia. *Latvijas Entomologs*, 42, 5-15.

Bonato, L., Voigtländer, K. & Minelli, A. (2012) *Algerophilus*, a neglected lineage of Western Mediterranean centipedes (Chilopoda: Geophilidae). *Zootaxa*, 3235, 23-34.

Bonato, L., Zapparoli, M. & Minelli, A. (2008) Morphology, taxonomy and distribution of *Diphyonyx* gen. n., a lineage of geophilid centipedes with unusually shaped claws (Chilopoda: Geophilidae). *European Journal of Entomology*, 105, 343-354.

Brölemann, H.-W. (1900) Voyage de M. Ch. Alluaud aux Iles Canaries (Nov. 1889 - Juin 1890). Myriapodes. *Mémoires de la Société zoologique de France*, 13, 431-452.

Brölemann, H.-W. (1901) Matériaux pour servir à une faune des myriapodes de France. *Le Feuille des Jeunes Naturalistes*, 31, 259-262.

Brölemann, H.-W. (1904) Chilopodes monégasques. I. Liste des chilopodes du territoire de la Principauté ou dans ses environs immédiats. II. Description de géophilides nouveaux. III. Un nouvel *Himantarium* monstrueux. *Bulletin du Musée Océanographique de Monaco*, 15, 1-15.

Brölemann, H.-W. (1908) La Haute Vallée de la Neste (Myriapodes). *Société d'Histoire naturelle et des Sciences biologiques et énergétiques de Toulouse*, *Bulletin*, 41, 57-67.

Brölemann, H.-W. (1909) A propos d'un système des Géophilomorphes. *Archives de Zoologie expérimentale et générale*, (5) 3, 303-340.

Brolemann, H.-W. (1924) Trois géophiliens (Myriapodes) nouveaux ou peu connus. *Bulletin de la Societe d'Histoire naturelle de Toulouse*, 52, 14-20.

Brolemann, H.-W. (1925) Races nouvelles de *Schizophyllum* algériens (Myriapodes-Diplopodes). *Bulletin de la Société d'Histoire naturelle de l'Afrique du Nord*, 16, 245-253.

Brolemann, H.-W. (1926) Myriapodes des Pyrénées-orientales. *Bulletin de la Société d'Histoire naturelle de Toulouse*, 54, 233-267.

Brolemann, H.-W. (1927) Trois Myriapodes Français nouveaux. *Bulletin de la Societe d'Histoire naturelle de Toulouse*, 56, 101-110.

Brolemann, H.-W. (1930) *Élements d'une Faune des Myriapodes de France. Chilopodes*. Imprimerie Toulosaine, Toulouse, 404 pp.

Brolemann, H.-W. (1932) Tableaux de détermination des chilopodes signalés en Afrique du Nord. *Bulletin de la Société d'Histoire naturelle de l'Afrique du Nord*, 23, 31-64.

Brölemann, H.-W. (1947) Catalogue des myriapodes chilopodes de la collection de l'Institut Scientifique Chérifien. *Bulletin de la Société des Sciences naturelles et physiques du Maroc*, 25-27, 172-182.

Brölemann, H.-W. & Ribaut, H. (1912) Essai d'une monographie des Schendylina (Myriapodes Géophilomorphes). *Nouvelles Archives du Muséum d'Historie naturelle, Paris*, (5) 4, 53-183.

Căpuşe, I. (1968) Contribution à l'étude des espèces appartenant aux genres *Insigniporus* Att. et *Pachymerium* C.L. Koch (Geophilomorpha Geophilidae). *Travaux du Muséum d'Histoire naturelle "Gr. Antipa"*, 8, 699-719.

Căpuşe, I. (1975) *Himantariella balearica* new species and *Pachymerium dragani* new species of Geophilomorpha from the Island of Majorca. *Travaux de l'Institut de Spéologie "Emile Racovitza"*, 14, 35-44.

Chalande, J. (1909) Nouvelle espèce pyrénéenne du genre *Geophilus*. *Feuille des Jeunes Naturalistes*, 39, 89-93.

Chalande, J. (1910) Nouvelle espèce française du genre *Geophilus*. *Bulletin de la Société d'Histoire naturelle et des Sciences biologiques et énergétiques de Toulouse*, 43, 87-92.

Chalande, J. & Ribaut, H. (1909) Études sur la systématique de la famille des Himantariidae (Myriapodes). *Archives de Zoologie expérimentale et générale*, (5) 1, 197-275.

Christian, E. (1996) Die Erdläufer (Chilopoda-Geophilida) des Wiener Stadtgebietes. *Verhandlungen der zoologisch-botanischen Gesellschaft in Österreich*, 133, 107-132.

Daday, J. (1889) *A Magyarországi Myriopodák Magánrajza*. Királyi Magyar Természettudományi Társulat, Budapest, 104 pp.

Dányi, L. (2006) Contribution to the Chilopoda fauna of the Muramureş (Romania). *Studia Universitatis Vasile Goldiş, Seria Şt. Vieţii*, 17, 43-46.

Dányi, L. (2007) *Geophilus oligopus* (Attems, 1895) a species new to the fauna of Romania and to the whole of the Carpathian Mountains. *Schubartiana*, 2, 39-48.

Dărăbanţu, C. (1972) Nouvelles espèces de géophilomorphes dans la faune de Roumanie. *Studia Universitatis Babeş-Bolyai. Series Biologia*, 17, 91-96.

Dărăbanţu, C. & Matic, Z. (1969) Genul *Clinopodes* C. Koch, 1847 (Geophilidae - Geophilomorpha) in fauna României. *Studia Universitatis Babeş-Bolyai. Serie Biologia*, 2, 101-107.

Demange, J.-M. (1959) Myriapodes Chilopodes de Madère (Mission de M. le Pr. A. Vandel). *Revue Française d'Entomologie*, 26, 157-166.

Demange, J.-M. (1961) Sur deux myriapodes gèophilomorphes des près salés d'Arcachon. *Procès-verbaux de la Société Linnéenne de Bordeaux*, 98, 3 pp.

Demange, J.-M. (1981) *Le Mille-pattes. Myriapodes*. Société Nouvelle des Éditions Boubée, Paris, 279 pp.

Dobroruka, L.J. (1957) Čtyři stonožky (Chilopoda) nové por faunu ČSR. *Casopis Národního Musea. Oddíl prírodovedny*, 126, 156-158.

Dobroruka, L.J. (1977) Bulgarische Chilopoden in der Sammlung von Nationalmuseum Praha. *Věstník Československé Společnosti zoologické*, 41, 5-7.

Eason, E.H. (1962) The chilopod genus *Nesoporogaster* Verhoeff. *Proceedings of the Zoological Society of London*, 138, 123-132.

Eason, E.H. (1964) *Centipedes of the British Isles*. F. Warne & Co., London, New York, 294 pp.

Enghoff, H. (1971) *Geophilus insculptus* en hidtil upåagtet dansk skolopender samt bemaerkninger om *G. proximus* C. L. Koch (Chilopoda). *Entomologiske Meddelelser*, 39, 119-121.

Faës, H. (1902) Myriopodes du Valais (Vallée du Rhône et vallées latérales). *Revue Suisse de Zoologie*, 10, 31-164.

Foddai, D. & Minelli, A. (1999) A troglomorphic geophilomorph centipede from Southern France (Chilopoda: Geophilomorpha: Geophilidae). *Journal of Natural History*, 33, 267-287.

Folkmanová, B. (1928) Chilopoda Republiky Ceskoslovenské. Dil I. *Fauna et Flora Cechoslovenica v Praze*, 3, 1-131.

Folkmanová, B. (1929) Eine neue Gattung der Unterfamilie der Geophilinen. *Zoologischer Anzeiger*, 84, 35-46.

Folkmanová, B. (1952) O nekterých zajímavých formách podrádu Geophilomorpha z Moravy a Slezska. *Prírodovedecký sborník Ostravského kraje*, 13, 179-192.

Folkmanová, B. (1956) On new forms of Geophilomorpha from Southern provinces of U.S.S.R. to the knowledge of the Myriapoda of U.S.S.R. [in Russian]. *Zoologicheskii Zhurnal*, 35, 1633-1646.

Folkmanová, B. & Dobroruka, L.J. (1960) Beitrag zur Kenntnis der Chilopoda der U.d.S.S.R. *Zoologicheskii Zhurnal*, 39, 1811-1818.

García Ruiz, A. & Serra, A. (2000) Nuevas citas de quilópodos (Myriapoda, Chilopoda) para la fauna de la Penísola Ibérica. *Boletin de la Asociacion española de Entomología*, 24 (3-4), 187-191.

Gregory, S. (1999) *Arenophilus peregrinus* Jones, 1989 in Cornwall: a centipede new to mainland Britain. *Bulletin of the British Myriapod Group*, 15, 28-29.

Gregory, S. & Barber, A. (2010) Observations of a population, including juveniles, of *Geophilus carpophagus* Leach, 1815, sensu stricto from Oxfordshire. *Bulletin of the British Myriapod Group*, 24, 2-15.

Haase, E. (1881) Schlesiens Chilopoden. II. Chilopoda Epimorpha. *Zeitschrift für Entomologie*, *Neue Folge*, 2, 66-92.

Hammer, P. (1931) Tusindben (Miriopoda). *Denmarks Fauna*, 35, 1-175.

Haswell, M., Enghoff, H. & Arthur, W. (2006) Further studies on *Geophilus carpophagus* (sensu lato) and a reinterpretation of the structure of its labrum. *Bulletin of the British Myriapod and Isopod Group*, 21, 2-7.

Horneland, E.O. & Meidell, B. (2009) Postembryonic development of *Strigamia maritima* (Leach, 1817) (Chilopoda, Geophilomorpha, Linotaeniidae) with emphasis on how to separate the different stadia. *Soil Organisms*, 81, 373-386.

Iorio, E. (2004) Contribution à la connaissance des Chilopodes des régions Centre, Île-de-France et Poitou-Charentes (Myriapoda). *Bulletin de la Société Linnéenne de Bordeaux*, 32, 235-255.

Iorio, E. (2005) A propos de quelques chilopodes recoltés en Île-de-France, en Picardie et dans le Centre (Myriapoda, Chilopoda). *Bulletin d'Arthropoda*, 23, 42-45.

Iorio, E. (2006) La faune des Chilopodes du Massif Armoricain. Biologie, liste préliminaire et détermination des espèces (Chilopoda). *Mémoires de la Société Linnéenne de Bourdeaux*, 7, 72 pp.

Iorio, E. (2008) Contribution à l'étude des chilopodes (Chilopoda) des Alpes-Maritimes, incluant une clé d'identification des lithobiomorphes Lithobiidae de Provence-Alpes-Côte d'Azur. *Bulletin de la Société Linnéenne de Provence*, 59, 127-190.

Jawlowski, H. (1949) Wije (Myriapoda) Bialowieskiego Parku Narodowego-Myriapoda of the National Park in Bialowieza. *Annales Universitatis Mariae Curie-Sklodowska*, Lublin, 4c, 309-323.

Jones, R.E. (1989) On a new species of centipede (Chilopoda Geophilomorpha) from the Isles of Scilly. *Journal of Natural History*, 23, 627-633.

Kaczmarek, J. (1969a) Beiträge zur Kenntnis bulgarischer Chilopoden. Teil I. *Bulletin de la Société des Amis des Sciences et des Lettres de Poznań, série D, Sciences Biologiques*, 9, 263-277.

Kaczmarek, J. (1969b) Beiträge zur Kenntnis bulgarischer Chilopoden. Teil II. Schendylidae. *Bulletin de la Société des Amis des Sciences et des Lettres de Poznań, série D, Sciences Biologiques*, 10, 99-109.

Kaczmarek, J. (1970) Beiträge zur Kenntnis bulgarischer Chilopoden. Teil III. *Bulletin de la Société des Amis des Sciences et des Lettres de Poznań, série D, Sciences Biologiques*, 11, 81-89.

Kaczmarek, J. (1972) Beiträge zur Kenntnis bulgarischer Chilopoden. Teil IV. *Bulletin de la Société des Amis des Sciences et des Lettres de Poznań, série D, Sciences Biologiques*, 12-13, 261-264.

Kaczmarek, J. (1979) Pareczniki (Chilopoda) Polski. *Wydawnictwo Naukowe Uniwersytetu im. A. Mickiewicza. Seria Zoologia*, 9, 1-99.

Kaczmarek, J. (1980) Pareczniki Chilopoda. *Katalog Fauny Polski*, Warszawa, 14, 43 pp.

Kaczmarek, J. (1981) Beiträge zur Kenntnis von *Strigamia perkeo* (Verhoeff, 1935) - (Chilopoda, Geophilomorpha). *Bulletin de la Société des Amis des Sciences et des Lettres de Poznań, série D, Sciences Biologiques*, 21: 133-138

Keay A.N. (1994) An investigation into the effect of environment on the number of pediferous segments in *Haplophilus subterraneus* Shaw - some preliminary results. *Bulletin of the British Myriapod Group*, 10, 35-37.

Kime, R.D. & Iorio, E. (2010) Centipedes and millipedes of Burgundy. *Bulletin of the British Myriapod & Isopod Group*, 24, 15-30.

Koch, C.L. (1863) *Die Myriapoden getreu nach der Natur abgebildet und beschrieben. 1-2*. H. W. Schmidt, Halle, 134+112 pp.

Koch, L. (1867) Zur Arachniden und Myriapodenfauna Süd-Europas. *Verhandlungen der zoologisch-botanischen Gesellschaft in Wien*, 17, 857-900.

Koch, L. (1882) Zoologische Ergebnisse von Excursionen auf den Balearen. II. Arachniden und Myriapoden. *Verhandlungen der zoologisch-botanischen Gesellschaft in Wien*, 31, 625- 678.

Koren, A. (1986) Die Chilopoden-Fauna von Kärnten und Osttirol. 1. Geophilomorpha, Scolopendromorpha. *Carinthia II*, 43, 1-85.

Latzel, R. (1880) *Die Myriopoden der Österreichisch-Ungarischen Monarchie. 1, Die Chilopoden*. Hölder, Wien, 228 pp.

Latzel, R. (1895) Beiträge zur Kenntniss der Myriopodenfauna von Madeira, den Selvages und den Canarischen Inseln. *Jahrbuch der Hamburgischen wissenschaftlichen Anstalten*, 12 (1894), 113-122.

Léger, L. & Duboscq, O. (1903) Recherches sur les Myriapodes de Corse et leurs parasites. *Archives de zoologie expérimentale et générale*, (4) 1, 307-358.

Lesniewska, M. (2012) *Morphological anomalies in* Haplophilus subterraneus *(Shaw, 1794) (Chilopoda: Geophilomorpha)*. Wydawnictwo Kontekt, Poznan, 208 pp.

Lesniewska, M. & Wojciechowski, J. (1992) *Haplophilus subterraneus* (Shaw 1794) (Chilopoda Geophilomorpha) - a representative of centipedes new for the fauna of Poland. *Przeglad Zoologiczny*, 361-4, 133-136.

Lewis, J.G.E. (1961) The life history and ecology of the littoral centipede *Strigamia* (= *Scolioplanes*) *maritima* (Leach). *Proceedings of the Zoological Society of London*, 137, 221-248.

Lewis, J.G.E. (1962) The ecology, distribution and taxonomy of the centipedes found on the shore in the Plymouth area. *Journal of the Marine Biological Association of the United Kingdom*, 42, 655-664.

Lewis, J.G.E. (1963) On *Clinopodes poseidonis* (Verhoeff) (Chilopoda: Geophilomorpha: Geophilidae) with a description of a new subspecies from the Red Sea littoral. *Annals and Magazine of natural History*, (13)6(B), 49-55.

Lewis, J.G.E. (1986) Centipedes of Saudi Arabia. *Fauna of Saudi Arabia*, 8, 20-30.

Lewis, J.G.E. (1994) On the true identity of *Geophilus sorrentinus* Attems (Chilopoda: Geophilomorpha). *Bulletin of the British Myriapod Group*, 10, 39-42.

Lewis, J.G.E., Jones, R.E. & Keay, A.N. (1988) On a new genus and species of centipede (Chilopoda Geophilomorpha Chilenophilidae) from the British Isles. *Journal of Natural History*, 22, 1657-1663.
Lewis, J.E.G. & Kime, R.D. (1988) Centipedes and millipedes from Finistère, Brittany. *Bulletin of the British Myriapod Group*, 5, 6-8.
Lignau, N. (1933) Einiges über die Myriapodenfauna der Ukraine. *Entomologicheskoe Obozrenie*, 25, 134-144.
Loksa, I. (1962) Über einige Chilopoden aus Österreich. *Opuscula Zoologica*, 4, 89-95.
Machado, A. (1952) Miriápodes de Portugal. Primeira parte: Quilópodes. *Broteria*, 21, 65-167.
Machado, A. (1953) Alguns miriápodes de Espanha. *Archivos del Instituto de Aclimatación, Almería*, 1, 77-92.
Manfredi, P. (1956) Miriapodi cavernicoli del Marocco, della Sardegna e del Piemonte. *Atti della Società Italiana di Scienze Naturali*, 95, 197-222.
Manfredi, P. (1957) I Miriapodi del Monte Pollino (Calabria) e considerazioni intorno ai Miriapodi dell'Italia meridionale. *Annuario dell'Istituto e Museo di Zoologia dell'Università di Napoli*, 9, 1-43.
Matic, Z. (1972) Clasa Chilopoda, Subclasa Epimorpha. *Fauna Republicii Socialiste România*, 6, 220 pp.
Matic, Z. (1985) Note sur les chilopodes du genre *Strigamia* Gray (Chilopoda: Geophilomorpha) de la Reserve Scientifique de « Retezat ». *Revue Roumaine de Biologie, Biologie Animale*, 30, 11-15.
Matic, Z. & Dărăbanţu, C. (1968) Contributo alla conoscenza dei Chilopodi Epimorfi (Chilopoda - Epimorpha) della fauna di Spagna. *Memorie del Museo civico di Storia naturale di Verona*, 16, 127-135.
Matic, Z. & Dărăbanţu, C. (1969) Contributo alla conoscenza del genere *Nesoporogaster* Verhoeff 1924 (Geophilomorpha). *Memorie del Museo civico di Storia naturale di Verona*, 16, 447-454.
Matic, Z. & Dărăbanţu, C. (1974) Contributions à la connaissance du genre *Thracophilus* Verhoeff (Geophilomorpha Himantariidae). *Bulletin de l'Institut de Zoologie et du Musée, Academie Bulgare des Sciences*, 40, 93-98.
Meinert, F. (1870) Myriapoda Musaei Hauniensis: bidrag til Myriapodernes morphologi og systematik. *Naturhistorisk Tidsskrift*, (3) 7, 1-128.
Mikos, M.Z. (1991) *Dicellophilus carniolensis* (C. L. Koch, 1847) kot predstavnik druzine Mecistocephalidae (superfam. Geophilomorpha). *Biološki Vestnik*, 39, 9-18.
Minelli, A. (1982a) Contributo alla revisione dei chilopodi geofilomorfi finora riferiti ai generi *Henia* e *Chaetechelyne* (Chilopoda Geophilomorpha). *Memorie della Società entomologica Italiana*, 60 (1981), 253-268.
Minelli, A. (1982b) Un nuovo chilopodo italiano, *Acanthogeophilus dentifer* n.g.n.sp. (Geophilomorpha Geophilidae). *Bollettino del Museo civico di Storia naturale di Verona*, 8 (1981), 235-240.
Minelli, A. (1983a) On Sardinian centipedes (Chilopoda). *Bollettino di Zoologia*, 49 (1982), 1-16.
Minelli, A. (1983b) Note critiche sui Chilopodi della Sardegna. *Lavori della Società Italiana di Biogeografia*, 8 (1980), 401-416.
Misioch, M. (1978) Variation of characters in some geophilid chilopods. *Abhandlungen und Verhandlungen des naturwissenschaftlichen Vereins in Hamburg*, N. F., 21-22, 55-62.
Misioch, M. (1979) Notes on the taxonomy of central European Geophilomorpha. *In:* Camatini, M. (Ed.), *Myriapod Biology*, Academic Press, London, pp. 83-94.
Newport, G. (1844) A list of the species of Myriapoda order Chilopoda contained in the cabinets of the British Museum, with synoptic descriptions of forty-seven new species. *Annals and Magazine of natural History*, (1) 13, 94-101.
Palmén, E. (1949) The Chilopoda of Eastern Fennoscandia. *Annales Zoologici Societatis Zoologicae-Botanicae Fennicae*, 13, 1-54.
Pocock, R.I. (1891) Descriptions of some new Geophilidae in the collections of the British Museum. *Annals and Magazine of natural History*, (6) 8, 215-227.
Porat, C.O. von (1871) Om några Myriopoder från Azorerna. *Öfversigt af Kongliga Vetenskaps-Akademiens Förhandlingar*, 27 (1870)(7), 813-823.
Ribarov, G.K. (1987) *Henia angelovi* sp. n. (Chilopoda Geophilidae). Eine neue *Henia* Art aus Südostbulgarien. *Acta Zoologica Bulgarica*, 35, 86-89.
Ribaut, H. (1910) Races de *Stigmatogaster gracilis* (Mein.) [Myriop.]. *Archives de Zoologie expérimentale et générale*, (5) 5, 41-43.
Ribaut, H. (1911) Sur un genre nouveau de la sous-tribu des Ribautiina Bröl. (Myriapoda-Geophilomorpha). *Bulletin de la Société d'Histoire naturelle et des Sciences biologiques et énergétiques de Toulouse*, 43 (1910), 105-126.
Rothenbühler, H. (1899) Ein Beitrag zur Kenntnis der Myriapodenfauna der Schweiz. *Revue Suisse de Zoologie*, 6, 199-210.
Salinas, J.A. (1990) Contribución al conocimiento de los quilópodos de Navarra (Myriapoda: Chilopoda). *Publicaciones de Biologia de la Universidad de Navarra, Serie zoologica*, 20, 1-70.
Silvestri, F. (1895) Contribuzione alla conoscenza dei Chilopodi, Symphyli, Pauropodi e Diplopodi dell'Umbria e del Lazio. *Bollettino della Società Romana per gli Studi zoologici*, 3, 191-201.
Silvestri, F. (1896a) Nuovi Diplopodi e Chilopodi dell'Italia settentrionale. *Bollettino dei Musei di Zoologia ed Anatomia comparata della Regia Università di Torino*, 11, 1-5.
Silvestri, F. (1896b) Una escursione in Tunisia (Symphyla, Chilopoda, Diplopoda). *Naturalista Siciliano*, N.S., 1, 143-161.
Silvestri, F. (1898a) Contributo alla conoscenza dei Chilopodi e Diplopodi della Sicilia. *Bollettino della Società entomologica italiana*, 29, 233-261.
Silvestri, F. (1898b) Contributo alla conoscenza dei Chilopodi e Diplopodi dell'isola di Sardegna. *Annali del Museo civico di Storia naturale di Genova*, (2) 18, 680-693.
Silvestri, F. (1907) Neue und wenig bekannte Myriopoden des naturhistorischen Museums in Hamburg (1. Teil). *Mitteilungen aus dem naturhistorischen Museum in Hamburg*, 2, 229-257.
Simaiakis, S. (2009) Relationship between intraspecific variation in segment number and geographic distribution of *Himantarium gabrielis* (Linné, 1767) (Chilopoda: Geophilomorpha) in Southern Europe. *Soil Organisms*, 81, 359-372.
Simaiakis, S.M, Iorio, E., Djursvoll, P., Meidell, B.A., Andersson, G. & Kirkendall, L.R. (2010) A study of the diversity and geographical variation in numbers of leg-bearing segments in centipedes (Chilopoda: Geophilomorpha) in north-western Europe. *Biological Journal of the Linnean Society*, 100, 899–909.
Simaiakis, S., Zapparoli, M., Minelli, A. & Bonato, L. (2013) The centipede fauna (Chilopoda) of the island of Cyprus, with one new lithobiomorph species. *Zootaxa*, 3647, 279-306.
Snellen van Vollenhoven, S.C. (1860) *Natuurlijke historie van Nederland. De dieren van Nederland. Overzigt der gelede dieren*. A.C. Kruseman, Haarlem, 2 vol., 558 pp.

Sograff, N.J. (1883) Materials toward the knowledge of the embryonic development of *Geophilus ferrugineus* and *Geophilus proximus* [in Russian]. *Izvêstya Imperatorskago Obshchestva Lyubitelei Estestvoznaniya, Antropologii i Etnografii pri Imperatorskom Moskovskom Universitete*, 2, 1-77.
Spelda, J. (1991) Zur Faunistik und Systematik der Tausendfüßler (Myriapoda) Südwestdeutschlands. *Jahrbuch der Gesellschaft für Naturkunde*, Württemberg, 146, 211-230.
Sseliwanoff, A. (1884) Materialii k isyceniyn russkih tyisiachenogich (Myriapoda). *Horae Societatis Entomologicae Rossicae*, 18 (1883-1884), 69-121.
Stavropoulos, G. & Matic, Z. (1990) Nouvelles contributions à la faune des chilopodes (Chilopoda) de Grèce. II. *Biologia Gallo-Hellenica*, 17, 37-47.
Stoev, P. (2002) *A catalogue and key to the centipedes (Chilopoda) of Bulgaria*. Pensoft, Sofia, 103 pp.
Titova, L.P. (1972) Novuie vidui roda *Escaryus* Cook & Collins (Schendylidae, Chilopoda). *In*: *Ekologiya Pochvennuich bespozvonochnuich*, Moskva, pp. 94-119.
Turk, F.A. (1947) Myriapodological notes. II. *Northwestern Naturalist*, Arbroath, 22, 226-234.
Turk, F.A. (1955) The Myriapoda of Dr. Cloudsley-Thompson's expedition to the Tunisian desert. *Annals and Magazine of natural History*, (12) 8, 277-284.
Vadell, M. & Pons, G.X. (2009) Aportaciones al conocimiento de los Quilòpodos (Chilopoda; Geophilomorpha) de la Serra de na Borguesa (Mallorca, Islas Baleares). *Bolletí de la Societat d'Història natural de les Balears*, 52, 169-182.
Verhoeff, K.W. (1895) Beiträge zur Kenntnis paläarktischer Myriopoden. (II. Aufsatz: Über mitteleuropäischen Geophiliden). *Archiv für Naturgeschichte*, 61, 346-356.
Verhoeff, K.W. (1896) Geophiliden und Scolopendriden aus Portugal und Tabelle europäischer *Geophilus*-Arten. *Zoologischer Anzeiger*, 19, 74-79, 81-89.
Verhoeff, K.W. (1898) Beiträge zur Kenntnis paläarktischen Myriopoden. VI. Über paläarktische Geophiliden. *Archiv für Naturgeschichte*, 64, 335-362.
Verhoeff, K.W. (1900) Über *Schendyla* und *Pectinunguis*. *Zoologischer Anzeiger*, 23, 483-486.
Verhoeff, K.W. (1901) Beiträge zur Kenntnis paläarktischer Myriopoden. XVI. Aufsatz: Zur vergleichenden Morphologie, Systematik und Geographie der Chilopoden. *Abhandlungen der Leopoldinisch-Carolinischen Deutschen Akademie der Naturforscher*, 77, 369-465.
Verhoeff, K.W. (1902) Über Chilopoden von Südsteiermark, Krain und Kroatien. *Sitzungs-Berichte der Gesellschaft naturforschender Freunde zu Berlin*, 1902, 90-93.
Verhoeff, K.W. (1908) Chilopoda. *In:* Bronn, H.G. (Ed.), *Klassen und Ordnungen des Tierreichs*, Akademische Verlagsgesellschaft, Leipzig, 5, 725 pp.
Verhoeff, K.W. (1924) Über Myriapoden von Mallorca und Ibiza. *Entomologisk Tidskrift*, 45, 99-109.
Verhoeff, K.W. (1925) Mediterrane Chilopoden und Notiz zur Periodomorphose der Juliden. *Zoologischer Anzeiger*, 64, 63-80.
Verhoeff, K.W. (1926) Zwei neue Geophilomorphen-Gattungen aus Thracien und Mexiko. *Zoologischer Anzeiger*, 69, 97-105.
Verhoeff, K.W. (1928) Geophilomorphen-Beiträge und eine *Lithobius*-Form. *Mitteilungen aus dem zoologischen Museum in Berlin*, 14, 228-286.
Verhoeff, K.W. (1930) Über einige von Herrn Dr. C. R. Boettger in Italien gesammelten Diplopoden, Chilopoden und Isopoden. *Mitteilungen aus dem zoologischen Museum in Berlin*, 16, 566-573.
Verhoeff, K.W. (1931) Chilopoden der Insel Elba (Isopoden). *Zoologischer Anzeiger*, 95, 302-312.
Verhoeff, K.W. (1934) Beiträge zur Systematik und Geographie der Chilopoden. *Zoologische Jahrbücher, Abteilung für Systematik, Ökologie und Geographie der Tiere*, 66, 1-112.
Verhoeff, K.W. (1935a) Quer durch Schwarzwald und schweizerischen Jura (Chiemgau), Chilopoden. *Verhandlungen des naturwissenschaftlichen Vereins in Karlsruhe*, 29, 181-208.
Verhoeff, K.W. (1935b) Über *Scolioplanes* (Chilopoda). *Zoologischer Anzeiger*, 111, 10-23.
Verhoeff, K.W. (1937a) Über Chilopoden des alemannischen Gaues. *Berichte der naturforschenden Gesellschaft zu Freiburg*, 35, 196-210.
Verhoeff, K.W. (1937b) Chilopoden von Cherso. *Abhandlungen und Berichte aus dem Museum für Naturkunde und Vorgeschichte und dem naturwissenschaftlichen Verein zu Magdeburg*, 6, 305-315.
Verhoeff, K.W. (1938) Chilopoden-Studien, zur Kenntnis der Epimorphen. *Zoologische Jahrbücher, Abteilung für Systematik, Ökologie und Geographie der Tiere*, 71, 340-385.
Verhoeff, K.W. (1939) Streitfüge durch Spessart, Odenwald und Hardt: Chilopoda. *Berichte der Naturforschenden Gesellschaft zu Freiburg im Breisgau*, 36, 225-243.
Verhoeff, K.W. (1940) Chilopoden von Kärnten und Tauern, ihre Beziehungen zu europäischen und mediterranen Ländern und über allgemeine geographische Verhältnisse. *Abhandlungen der Preussischen Akademie der Wissenschaften, mathematisch-naturwissenschaftliche Klasse*, 5, 3-39.
Verhoeff, K.W. (1941) Asyanin zoogeografiyasi ve hayvan sistematige hakkinda. Asiatische Beiträge. *Istanbul Üniversitesi Fen Fakültesi Mecmuasi*, 6, 85-117.
Verhoeff, K.W. (1942) Zur Kenntnis Mediterraner Chilopoden besonders der Insel Ischia. *Zeitschrift für Morphologie und Ökologie der Tiere*, 38, 483-525.
Verhoeff, K.W. (1943a) Über Chilopoden der Insel Korsika. *Zoologischer Anzeiger*, 143, 1-20.
Verhoeff, K.W. (1943b) Chilopoden der Insel Kapri und der Sorrentinischen Halbinsel. *Zoologischer Anzeiger*, 141, 61-93.
Verhoeff, K.W. (1943c) Neuer Beitrag zur Kenntnis der Chilopoden der Insel Ischia. *Zoologischer Anzeiger*, 142, 62-83.
Verhoeff, K.W. (1943d) Über Chilopoden aus westbalkanischen Höhlen. *Zeitschrift für Karst- und Höhlenkunde*, 1942-1943, 133-152.
Verhoeff, K.W. (1945) Asya hayvanlari ve Zoogeografiyasi hakkinda. Asiatische Beiträge VIII. *Istanbul Üniversitesi Fen Fakültesi Mecmuasi*, (B) 9 (1944), 307-347.
Verhoeff, K.W. (1951) Diplopoda, Chilopoda und Isopoda terrestria vom Mt. Soratte in Latium. *Zoologische Jahrbücher, Abteilung für Systematik, Ökologie und Geographie der Tiere*, 80, 205-255.
Zalesskaja, N.T., Titova, L.P. & Golovatch, S.I. (1982) The myriapod fauna (Myriapoda) of the Moscow area [in Russian]. *In:* Ghilarov, M.S. (Ed.), *Soil Invertebrates of the Moscow Region*. Nauka, Moskow, pp. 179-200.
